# Q Fever in France, 1985–2009

**DOI:** 10.3201/eid1703.100882

**Published:** 2011-03

**Authors:** Diane Frankel, Hervé Richet, Aurélie Renvoisé, Didier Raoult

**Affiliations:** Author affiliation: Université de la Méditerranée, Marseille, France

**Keywords:** Q fever, Coxiella burnetii, bacteria, seasonality, survey, geographic repartition, endocarditis, outbreak, France, synopsis

## Abstract

To assess Q fever in France, we analyzed data for 1985–2009 from the French National Reference Center. A total of 179,794 serum samples were analyzed; 3,723 patients (one third female patients) had acute Q fever. Yearly distribution of acute Q fever showed a continuous increase. Periodic variations were observed in monthly distribution during January 2000–December 2009; cases peaked during April–September. Q fever was diagnosed more often in patients in southeastern France, where our laboratory is situated, than in other areas. Reevaluation of the current positive predictive value of serologic analysis for endocarditis was performed. We propose a change in the phase I (virulent bacteria) immunoglobulin G cutoff titer to >1,600. Annual incidences of acute Q fever and endocarditis were 2.5/100,000 persons and 0.1/100,000 persons, respectively. Cases and outbreaks of Q fever have increased in France.

Q fever is a worldwide zoonosis caused by the intracellular bacterium *Coxiella burnetii* (*1*). Cases of Q fever in the United States reported during the second Gulf War in Iraq have raised new interest in this disease; Q fever is generally considered a forgotten infectious disease in developing countries (*2*). In a study conducted in France during 1985–1998, the main symptoms of Q fever were classified. This study also reported monthly incidence; peaks in April, May, and June were also identified (*3*). Furthermore, large outbreaks of Q fever in the Netherlands (*4*) and in a school in Israel (*5*) during the past 3 years have brought attention to the frequency of Q fever.

Q fever is primarily transmitted by cattle, goats, and sheep (*6*). Humans are infected by aerosols from parturient fluids of infected livestock. *C*. *burnetii* is resistant to environmental conditions and can survive for several weeks or months in areas where livestock are present. These bacteria can also be transmitted by wind (*7*).

The main characteristic of Q fever is its clinical polymorphism. Acute Q fever is defined as primary infection with *C. burnetii*, and <60% of infected patients may be asymptomatic. However, Q fever can manifest as an influenza-like syndrome, pneumonia, or hepatitis, and 2% of patients with acute disease are hospitalized. Chronic Q fever is defined as an infection that lasts 6 months and has a phase I (virulent bacteria) immunoglobulin (Ig) titer >800. The most common form of chronic Q fever includes endocarditis (73%). Its estimated prevalence among cases of Q fever is 2%–5% (*1*). This pathology often occurs when patients have underlying valvulopathy. Osteomyelitis, vascular infections, and chronic hepatitis have also been described (*2**,**8*). Serologic profiles of patients with acute and chronic Q fever differ. In the acute form, IgM against phase I and phase II (avirulent bacteria) Q fever is observed. In the chronic phase, high levels of IgG against both phases are observed (*9*).

Because symptoms of Q fever are nonspecific (*10*), its diagnosis is still based on serologic analysis and varies with the attention of the attending physician, true prevalence of the disease, and quality of the diagnostic tests (*2*). The definitions of acute and chronic Q fever are based on a previous study conducted when testing for Q fever was performed infrequently (*10*). The French National Reference Center (NRC) database was created in 1985. Because Q fever seemed to be rapidly reemerging in France, we conducted a study that focused on potential causes, seasonality, geographic distribution, and outbreaks of the disease.

## Patients

At the NRC, specimens from patients with suspected Q fever are collected routinely. We used these samples for serologic analysis. In cases of endocarditis, heart valves were tested by histological analysis, immunohistochemical analysis, PCR, or cell culture. Serum samples were transported at ambient temperature and valve samples were transported at –80°C. Since 1985, all serum samples received have been screened by immunofluorescent assay by using a large panel of antigens, including those from *C. burnetii*, *Rickettsia* spp., *Bartonella* spp., and *Francisella tularensis*. When antibody titers indicated infection with *C. burnetii*, monitoring was proposed with a serologic control 3 weeks after the first sample. However, for this study, only the first positive serum sample was used to determine whether Q fever showed a seasonal distribution and to evaluate the incidence of Q fever. For each patient, geographic origin, sex, age, and test results were added to 1 database.

Every year, the NRC provides an activity report in which annual data are analyzed. For Q fever, the number of positive serum samples and the total number of serum tested are included. The report describes the number of acute and chronic Q fever cases for the year.

Q fever seasonality and geographic distribution during January 2000–December 2009 were analyzed. We also classified all patients during 2009 on the basis of 2 criteria: 1) first positive serologic result in 2009 and 2) clinical status of the patient in the database. This classification was performed to evaluate incidence of endocarditis according to serologic titer and modified Duke Criteria. Patients were classified as having definite or possible endocarditis (*11*).

## Serologic Analysis

Antigens were prepared for testing of phase I and II samples as described (*10*). Serum samples were first screened at dilutions of 1:25, 1:50, and 1:100 by using an indirect immunofluroescent antibody (IFA) test. Serum samples positive for total antibodies against Q fever at 1:100 dilution were tested by IFA to determine the IgG, IgM, and IgA titers for phases I and II. Serum with a phase II IgG titer >200 and a phase II IgM titer >50 was predictive for acute Q fever. This serologic criterion was used to define acute Q fever. If a phase I IgG titer was >800, chronic Q fever was suspected, which depended on other clinical findings. A low level of IgG is considered evidence of past infection (*12*).

## Statistical Analysis

Since 1991, serologic results have been entered into our laboratory database and can be extracted as Excel (Microsoft, Redmond, WA, USA) files. Excel sheets were analyzed by using PASW Statistics software version 17.0 (SPSS Inc., Chicago, IL, USA). Seasonality of acute Q fever during January 2000–December 2009 was studied by using Expert Modeler (SPSS Inc.). This software identified the best model to use to analyze the data. Dependent variable series were also analyzed by using Expert Modeler, which automatically generates the best-fitting seasonal or nonseasonal model. This software also provides a better understanding of the data and predictions for future points in the series. Stationary R^2^ goodness was used to measure the level of compliance of the data compared with the theoretical model. The Ljung-Box test (http://www.itl.nist.gov/div898/software/dataplot/refman1/auxillar/ljungbox.htm) was used to test the null hypothesis that autocorrelations of the residual time series were equal to 0. Statistical analyses of geographic distribution, mean age, and sex were conducted by using Epi Info version 6 (Centers for Disease Control and Prevention, Atlanta, GA, USA).

## Evolution of Q Fever

**Figure 1:**
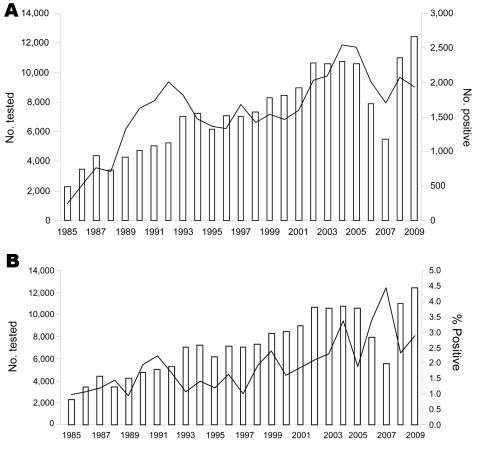
Serum samples tested for Q fever, France, 1985–2009. A) Black line indicates no. positive. B) Black line indicates % positive for acute Q fever.

During January 1985–December 2009, a total of 179,794 serum samples were analyzed (Table 1, Figure 1); 39,472 (30%) were positive, i.e., positive for antibodies at the first screening at a 1:100 dilution; 3,723 patients had acute Q fever (phase II IgG titer >200 and phase II IgM titer >50), and 1,675 had chronic Q fever (phase I IgG titer >800). The number of serum samples analyzed increased each year (except in 2006 and 2007), from 2,290 in 1985 to 12,443 in 2009. The number of positive serum samples also increased; peaks occurred in 1992 and in 2004. In 2004, the increase in serum samples positive for Q fever showed a correlation with increases in acute and chronic Q fever; 114 cases of acute Q fever were diagnosed in the Marseille area. Of the patients with acute Q fever, we had data for 919 (33.8%) female patients and 1,799 (66.2%) male patients.

**Table 1:** Serum samples tested for Q fever, by year, France*

Year	No. positive/ no. tested (%)	No. positive	
		Acute disease	Chronic disease
1985	239/2,290 (10.4)	22	8
1986	518/3,464 (15.0)	37	8
1987	767/4,361 (17.6)	52	12
1988	706/3,403 (20.7)	49	12
1989	1,316/4,258 (30.9)	40	18
1990	1,621/4,720 (34.3)	92	22
1991	1,737/5,028 (34.5)	112	20
1992	2,011/5,249 (38.3)	89	29
1993	1,815/7,020 (25.9)	76	26
1994	1,472/7,222 (20.4)	102	24
1995	1,359/6,171 (22.0)	73	34
1996	1,326/7,101 (18.7)	116	28
1997	1,683/7,050 (23.9)	70	35
1998	1,420/7,340 (19.3)	140	37
1999	1,537/8,296 (18.5)	199	32
2000	1,460/8,444 (17.3)	135	32
2001	1,589/8,974 (17.7)	167	38
2002	2,029/10,639 (19.1)	224	55
2003	2,094/10,588 (19.8)	242	52
2004	2,544/10,742 (23.7)	360	70
2005	2,514/10,597 23.7)	199	100
2006	2,010/7,891 (25.5)	266	237
2007	1,700/5,522 (30.8)	244	278
2008	2,073/10,981 (18.9)	256	263
2009	1,933/12,443 (15.5)	361	205
Total	39,472/179,794 (21.9)	3,723	1,675

*Acute disease, phase II (avirulent bacteria) immunoglobulin (Ig) G titer >200 and phase II IgM titer >50; chronic disease, phase I (virulent bacteria) IgG titer >800.

**Figure 2:**
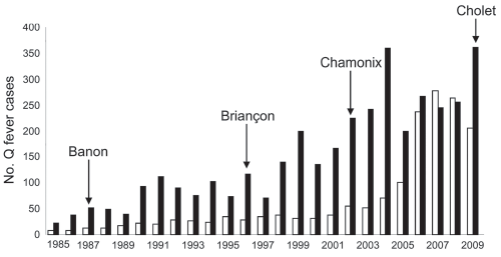
Cases of chronic (white bars) and acute (black bars) Q fever, France, 1985–2009. Locations where outbreaks were reported are indicated by arrows.

Cases of acute Q fever increased continuously during 1985–2009 (Figure 2). The number of patients with serologic results for Q fever was stable; <100 cases were detected per year until 2004. Subsequently, >200 cases were diagnosed every year because physicians obtained more samples from patients for follow-up of acute infection (Figure 2).

**Figure 3:**
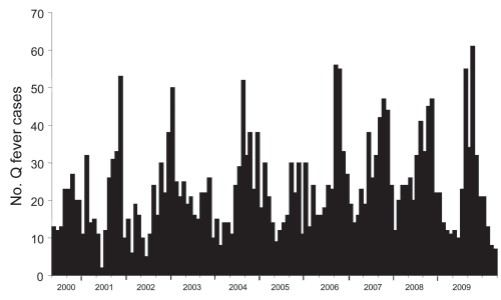
Seasonality of acute Q fever cases, France, 2000–2009.

The monthly distribution of acute Q fever during January 2000–December 2009 was determined. Variations in number of acute Q fever cases periodically occurred (Figure 3). Infections peaks occurred every year during April–September. Lower infection rates occurred during October–January. No peaks were observed in 2003 or 2005, which had <30 cases per month. The highest value was observed in June 2009 and was related to the outbreak in Cholet and to cases in Bouches du Rhône.

The Autoregressive Integrated Moving Average model selected by the Expert Modeler found a good correlation between observed and predicted values and also demonstrated an ability to predict peaks of infections. The stationary R^2^ goodness-of-fit obtained with this model indicated that 49% of the variation in the number of acute Q fever cases was explained by the model (i.e., the month, which is the variable in our model, explained 49% of the fluctuation in the rate of Q fever rate). However, the Ljung-Box test value of 0.034 suggested that patterns in the observed series were not accounted for in the model.

**Figure 4:**
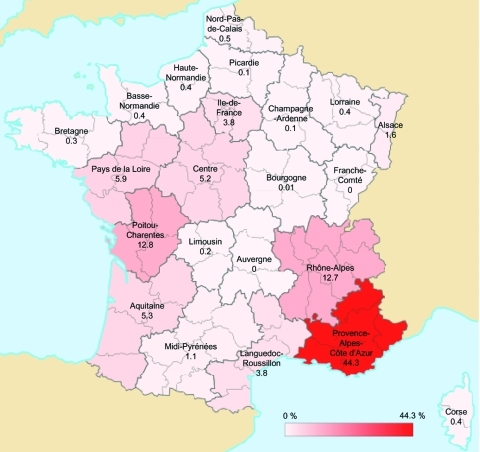
Geographic distribution of acute Q fever cases, France, 2000–2009. Values and scale bar indicate % prevalence.

Data regarding geographic distribution were obtained during January 1, 2000–December 31, 2009 (Figure 4). Geographic origin was known for 2,048 of 2,798 patients; data for 750 (26%) patients were missing. A total of 907 patients with Q fever were living in Provence Alpes Côte d’Azur, the region in which the NRC is located. The incidence of Q fever in this region was 19 cases/1 million inhabitants/y. Rhône-Alpes and Poitou-Charentes each had 12.7% of the cases. Incidence of cases in other regions did not exceed 6% (Figure 4).

Patient age was recorded for 2,658 patients during 2000–2009. The mean ± SD age was 46.2 ± 16.8 years, and the median age was 45 years (range 0–93 years). The mean ± SD age was 44.9 ± 18 years (median 43 years, range <1–90 years) for female patients and 46.8 ± 16.14 years (median 46 years, range 1–93 years) for male patients. The difference in mean age between male and female patients was significant (p = 0.0072).

In 2009, we obtained clinical information for 325 patients with positive serologic results. For this study, we classified the patient only if we obtained echocardiographic results for each patient. This process resulted in data for 172 patients according to the modified Duke Criteria (*11*). We classified these 172 patients according to their phase I IgG titers and identified 4 possible cases of endocarditis and 0 definite cases of endocarditis in patients whose phase I IgG titers were <800. For patients with phase I IgG titers ≥800, 1,600, 3,200 and 6,400, the numbers of definite cases of endocarditis were 41, 32, 24, and 17, respectively, and the numbers of possible cases of endocarditis were 11, 9, 3, and 1, respectively. The positive predictive values (PPVs) of IgG1 serologic results for possible or definite endocarditis were 37%, 59%, 57%, and 75% at titers of 800, 1,600, 3,200 and >6,400, respectively (Table 2).

**Table 2:** PPV of Q fever phase I IgG titers for patients with endocarditis, France, 2009*

Characteristic	Titers			
	>800	>1,600	>3,200	>6,400
No. with definite endocarditis	41	32	24	17
No. with possible endocarditis	11	9	3	1
Total	141	88	47	24
PPV, % (definite or possible endocarditis)	37	59	57	75

*PPV, positive predictive value; phase I, virulent bacteria; Ig, immunoglobulin.

## Reemergence?

In this study, we demonstrated an increase in the yearly incidence of acute Q fever. Although we observed a clear trend of increased testing (Table 1), the data also showed an increase in the percentage positive among the samples being tested, increasing from 1% in 1989 to 3%–4% in 2005–2009 (Figure 1). These findings suggest several interpretations of this increased incidence. One interpretation could be improved diagnostic capability caused by development and availability of commercial diagnostic tests. At this stage, only ELISAs (Panbio, Sinnamon Park, Queensland, Australia, and Virion/Serion, Würzburg, Germany) and immunofluorescent assays (Focus Diagnostics, Cypress, CA, USA) are available. In our laboratory, in addition to PCR, an in-house method based on an IFA has been used for Q fever diagnosis since 1985. The continuity of our techniques enables comparison of results. Ake et al. demonstrated heterogeneous serologic results that depended on the method used (*13*). They focused on the necessity of having a reference laboratory to reduce potential interlaboratory and interassay variability.

Despite the increasing number of serum samples received by the NRC, our collection was insufficient to accurately determine the incidence of Q fever in France. In the United States, surveillance for this disease has historically been limited, and only 436 cases were reported during 1978–1999. In 1999, Q fever became a reportable disease in the United States, and the number of cases increased by 250% during 2000–2004 because of improved recognition and reporting (*14*). This study shows the need for national surveillance and confirms that lack of interest in the disease has consequences when incidence is determined.

Outbreaks of Q fever in France contributed to increases in incidence in 1987, 1996, 2002–2003, and 2009. However, incidence can be determined only if physicians are aware of Q fever. In 1987, forty cases of acute Q fever were diagnosed at a psychiatric institution in Banon, France (*15*). This outbreak affected patients and staff who worked on a farm where goats were raised for milk and cheese production. An outbreak was also reported in 1996 in Briançon, France, where 29 cases of acute Q fever were identified (*16*). Analysis of risk factors in a case–control study suggested that transmission resulted from airborne contaminated sheep waste, which had been left uncovered in a slaughterhouse area. Another outbreak was detected in 2002 in Chamonix, France, in which 101 patients were diagnosed with acute Q fever during August 30, 2002–July 31, 2003 (*17*). The most recent outbreak in France was at an abattoir in Cholet in 2009, in which 50 cases of acute Q fever were detected and confirmed (D. Raoult, unpub. data).

Outbreaks in the Netherlands during the past 3 years (182, 1,000, and 2,361 cases in 2007, 2008, and 2009, respectively) have emphasized the need to detect Q fever in livestock, particularly in goats (*4*). When infected animal fetuses are aborted, massive amounts of microbes are released. Goats and other infected animals are predominantly asymptomatic, which makes Q fever difficult to detect. Investigations in the Netherlands showed that outbreaks in 2007, 2008, and 2009 started in ≈2005. Measures taken in this country included massive vaccination of goats with an animal vaccine produced by CEVA (Libourne, France). Although this vaccine does not prevent all infections and is not effective in infected animals, it does prevent most abortions. Therefore before vaccination, farmers must kill all pregnant goats on affected farms, regardless of the vaccination status of the animals. However, although outbreaks were a factor in the increase in Q fever, they were likely not the sole cause of increases in recent years.

Another reason for the increase in incidence of Q fever was increased interest of physicians in this disease. Results from a survey showed that the highest incidence of this disease in France was in Bouches du Rhône, where our laboratory is located. The proximity of the NRC has been suggested to influence the number of cases detected in this area; the annual incidence in our area (19 cases/1 million persons) was much higher than in the rest of France (3 cases/1 million persons). However, other reasons may explain changes in the geographic distribution of this disease.

Van der Hoek et al. reported the distribution of Q fever in humans in the Netherlands in 2009; a total of 59% of the cases were in persons who lived within a 5-km radius of an infected dairy sheep or dairy goat farm (12% of the population lived in these areas). The incidence was 69 cases per 100,000 persons in those who lived within a 5-km radius of infected livestock and 6 cases per 100,000 persons in those who lived outside the 5-km radius (*18*). Angelakis and Raoult reported that contaminated aerosols are the major mechanism for transmission of *C*. *burnetii* to humans (*19*). Data for 2008 from the French Agriculture Ministry (www.agreste.agriculture.gouv.fr) indicate that regions with the highest incidence of acute Q fever are regions with the highest number of cattle, sheep, and goats. Provence Alpes Côte d’Azur, the region in which the largest number of cases was detected, ranks fourteenth among French regions for number of livestock. Rhône-Alpes and Poitou Charente rank twelfth and sixth, respectively. Midi-Pyrénées, which contains the largest number of livestock, accounted for only 1.1% of acute Q fever cases in France.

Analysis of the monthly distribution of acute Q fever showed seasonality of the disease. Several factors might account for this phenomenon. *C*. *burnetii* is present in aerosols of parturient fluids from infected animals (*1*). Tissot-Dupont et al. reported seasonal increases in April and June (*20*) that were not correlated with sheep birth, which occurs in October. This observation could be explained by an increased number of lambs killed for Easter. Tissot-Dupont et al. also reported the role of wind in transmitting *C*. *burnetii* (*7*); an increase in incidence of Q fever during winter of 1998–99 correlated with increased velocity of mistral (the wind in southern France). These authors reported fewer cases of Q fever occurred in mountainous areas than in plains (*20*). A study in the United Kingdom demonstrated the role of airborne transmission (*21*). Hellenbrand et al. analyzed cases of Q fever in Germany during 1962–1999 (*22*). They found an irregular cyclic incidence pattern, but did not distinguish between acute and chronic forms of the disease. They also reported a change in community outbreak seasonality in Germany: a marked decrease in winter and an increase in summer. These changes coincide with decreases in nomadic sheep farming. These authors concluded that the incidence of Q fever has increased in Germany.

The number of patients in France with a chronic Q fever serologic profile has increased since 2006. However, with an average of 67 cases of chronic Q fever detected per year during 1985–2009, we expected to identify 50% of the chronic disease cases in France. Our data are more comprehensive because the NRC is the only laboratory that analyzes the phase I antibodies against Q fever in France.

Criteria for diagnosis of chronic Q fever were established in 1994 (*10*). Before this study, the PPV was determined by using Bayes theorem, and patients with endocarditis were tested for Q fever. The PPV for phase I IgG titers of 800 and 1,600 was high (98.1% and 100%, respectively). When we analyzed data from 2009, the PPV for possible or definite Q fever endocarditis (Table 2) was 37% for patients with phase I IgG titers >800 and 75% for patients with phase I IgG titers >6,400. This difference between the 2 studies reflects the increasing number of samples tested. In 2009, we diagnosed 41 definite and 11 possible cases of endocarditis caused by *C*. *burnetii*. A study published in 2000 with NRC data for 1985–1998 reported 259 cases of Q fever endocarditis and a mean of 20 cases per year (*3*).

The serum collection at the NRC is more comprehensive than it was 10 years ago, which could explain the increase in the number of cases. We believe that the number of *C*. *burnetii* endocarditis cases diagnosed at the NRC represents 50%–80% of the Q fever endocarditis cases in France. These data confirm previous data from which the prevalence of Q fever endocarditis in the general population in France was estimated to be 1 case/1 million persons/y. Several studies have estimated that 3%–5% of symptomatic *C*. *burnetii* infections cause endocarditis (*1*). These data enabled us to estimate the minimum incidence of mild-to-severe acute Q fever to be 20 cases/1 million persons/y, which is similar to that observed in Provence-Alpes-Côte d’Azur (19 cases/1 million persons/y) but lower than that observed in Bouches du Rhône (40 cases/1 million persons/y in 2009). When no outbreak is observed, the incidence of cases diagnosed in France at the NRC reflects the true incidence of acute infections.

Q fever has been recognized since the 1930s. We believe that its increased frequency in recent years is a combination of an increase in the disease (reemergence), growing interest among physicians, better diagnosis in laboratories, and more complete data. Monthly analysis of the past decade demonstrates the seasonality of Q fever, which shows peaks during April–September in Europe. We propose a change in the phase I IgG cutoff titer for detection of Q fever endocarditis to >1,600; this change corresponds to a PPV of 59%. This adjusted cutoff value may increase detection of endocarditis in patients with a diagnosis of chronic Q fever. Finally, the number of identified epidemics in France and Europe is increasing. Increased surveillance of the disease is the only way to determine the effects of Q fever.

## References

[1] Maurin M, Raoult D. Q fever. Clin Microbiol Rev. 1999;12:518–53. 10515901 10.1128/cmr.12.4.518 PMC88923

[2] Raoult D. Reemergence of Q fever after 11 September 2001. Clin Infect Dis. 2009;48:558–9. 10.1086/596706 19191639

[3] Raoult D, Tissot-Dupont H, Foucault C, Gouvernet J, Fournier PE, Bernit E, Q fever 1985–1998. Clinical and epidemiologic features of 1,383 infections. Medicine (Baltimore). 2000;79:109–23. 10.1097/00005792-200003000-00005 10771709

[4] Enserink M. Infectious diseases. Questions abound in Q-fever explosion in the Netherlands. Science. 2010;327:266–7. 10.1126/science.327.5963.266-a 20075230

[5] Amitai Z, Bromberg M, Bernstein M, Raveh D, Keysary A, David D, A large Q fever outbreak in an urban school in central Israel. Clin Infect Dis. 2010;50:1433–8. 10.1086/652442 20415568

[6] Tissot-Dupont H, Raoult D. Q fever. Infect Dis Clin North Am. 2008;22:505–14. 10.1016/j.idc.2008.03.002 18755387

[7] Tissot-Dupont H, Amadei MA, Nezri M, Raoult D. Wind in November, Q fever in December. Emerg Infect Dis. 2004;10:1264–9. 15324547 10.3201/eid1007.030724 PMC3323349

[8] Botelho-Nevers E, Fournier PE, Richet H, Fenollar F, Lepidi H, Foucault C, *Coxiella burnetii* infection of aortic aneurysms or vascular grafts: report of 30 new cases and evaluation of outcome. Eur J Clin Microbiol Infect Dis. 2007;26:635–40. 10.1007/s10096-007-0357-6 17629755

[9] Fournier PE, Marrie TJ, Raoult D. Diagnosis of Q fever. J Clin Microbiol. 1998;36:1823–34. 9650920 10.1128/jcm.36.7.1823-1834.1998 PMC104936

[10] Dupont HT, Thirion X, Raoult D. Q fever serology: cutoff determination for microimmunofluorescence. Clin Diagn Lab Immunol. 1994;1:189–96. 7496944 10.1128/cdli.1.2.189-196.1994 PMC368226

[11] Fournier PE, Casalta JP, Habib G, Messana T, Raoult D. Modification of the diagnostic criteria proposed by the Duke Endocarditis Service to permit improved diagnosis of Q fever endocarditis. Am J Med. 1996;100:629–33. 10.1016/S0002-9343(96)00040-X 8678083

[12] Gozalan A, Rolain JM, Ertek M, Angelakis E, Coplu N, Basbulut EA, Seroprevalence of Q fever in a district located in the west Black Sea region of Turkey. Eur J Clin Microbiol Infect Dis. 2010;29:465–9. 10.1007/s10096-010-0885-3 20195671

[13] Ake JA, Massung RF, Whitman TJ, Gleeson TD. Difficulties in the diagnosis and management of a US service member presenting with possible chronic Q fever. J Infect. 2010;60:175–7. 10.1016/j.jinf.2009.09.010 19766138

[14] McQuiston JH, Holman RC, McCall CL, Childs JE, Swerdlow DL, Thompson HA. National surveillance and the epidemiology of human Q fever in the United States, 1978–2004. Am J Trop Med Hyg. 2006;75:36–40. 16837706 10.4269/ajtmh.2006.75.1.0750036

[15] Fishbein DB, Raoult D. A cluster of *Coxiella burnetii* infections associated with exposure to vaccinated goats and their unpasteurized dairy products. Am J Trop Med Hyg. 1992;47:35–40. 1636881 10.4269/ajtmh.1992.47.35

[16] Carrieri MP, Tissot-Dupont H, Rey D, Brousse P, Renard H, Obadia Y, Investigation of a slaughterhouse-related outbreak of Q fever in the French Alps. Eur J Clin Microbiol Infect Dis. 2002;21:17–21. 10.1007/s10096-001-0645-5 11913496

[17] Tissot-Dupont H, Vaillant V, Rey S, Raoult D. Role of sex, age, previous valve lesion, and pregnancy in the clinical expression and outcome of Q fever after a large outbreak. Clin Infect Dis. 2007;44:232–7. 10.1086/510389 17173223

[18] van der Hoek W, Dijkstra F, Schimmer B, Schneeberger PM, Vellema P, Wijkmans C, Q fever in the Netherlands: an update on the epidemiology and control measures. Euro Surveill. 2010;15:19520. 20350500

[19] Angelakis E, Raoult D. Q fever. Vet Microbiol. 2010;140:297–309. 10.1016/j.vetmic.2009.07.016 19875249

[20] Tissot-Dupont H, Torres S, Nezri M, Raoult D. Hyperendemic focus of Q fever related to sheep and wind. Am J Epidemiol. 1999;150:67–74. 10400556 10.1093/oxfordjournals.aje.a009920

[21] Wallensten A, Moore P, Webster H, Johnson C, van der Burgt G, Pritchard G, Q fever outbreak in Cheltenham, United Kingdom, in 2007 and the use of dispersion modelling to investigate the possibility of airborne spread. Euro Surveill. 2010;15:19521. 20350497

[22] Hellenbrand W, Breuer T, Petersen L. Changing epidemiology of Q fever in Germany, 1947–1999. Emerg Infect Dis. 2001;7:789–96. 10.3201/eid0705.010504 11747689 PMC2631891

